# Duration of breastfeeding, age at introduction of complementary foods and allergy-related diseases: a prospective cohort study

**DOI:** 10.1186/s13006-020-00352-2

**Published:** 2021-01-06

**Authors:** Louise Ekelund, Inga Gloppen, Torbjørn Øien, Melanie Rae Simpson

**Affiliations:** 1 Faculty of Medicine, Norwegian University of Science and Technology, NTNU, Trondheim, Norway; 2 Department of Public Health and Nursing, Norwegian University of Science and Technology, NTNU, Trondheim, Norway; 3 Clinic of Laboratory Medicine, St Olavs Hospital, Trondheim, Norway

**Keywords:** Breastfeeding, Complementary food, Allergy, Asthma, Wheeze, Eczema, Allergic rhinoconjunctivitis, Prevention

## Abstract

**Background:**

The influences of breastfeeding and infant diet in the prevention of allergy-related diseases are uncertain and many of the studies conducted on the topic are limited by methodological challenges. Our aim was to assess whether the duration of breastfeeding and age at complementary food introduction affected the prevalence of asthma, wheeze, allergic rhinoconjunctivitis (ARC) and eczema at two and six years of age.

**Methods:**

We used information gathered between 2000 and 2014 through questionnaires in the Prevention of Allergy among Children in Trondheim (PACT) study, a prospective cohort study in Trondheim, Norway. The current study includes 6802 children who submitted questionnaires detailing breastfeeding duration and or age at introduction to complementary foods, as well as at least one of the child health questionnaires completed at two and six years of age. Adjusted odds ratios (aORs) were calculated for each combination of exposure and outcomes and sensitivity analyses were performed to assess the possible influence of recall bias and reverse causality.

**Results:**

The mean duration of breastfeeding was 11 months (SD 5.6) in this study population and 5695 of 6796 (84%) infants had been breastfed for at least 6 months. We did not find any conclusive preventative effect of longer breastfeeding on parental reported doctor-diagnosed asthma, aOR 0.79 (95% CI 0.51, 1.21). However, at 6 years of age we observed a reduction in the less strictly defined outcome wheeze, aOR 0.71 (95% CI 0.53, 0.95). Longer breastfeeding was associated with a reduced risk of ARC at 2 years, aOR 0.65 (95% CI 0.49, 0.86), with a continued protective trend at 6 years, aOR 0.77 (95% CI 0.58, 1.04).

**Conclusions:**

Longer breastfeeding resulted in a reduced risk of wheeze and a trend towards a protective effect on ARC up until school age. No conclusive associations were seen between the duration of breastfeeding or age at introduction to complementary foods and prevention of asthma, wheeze, ARC and eczema.

**Trial registration:**

The trial is registered in Current Controlled Trials as ISRCTN28090297.

**Supplementary Information:**

The online version contains supplementary material available at 10.1186/s13006-020-00352-2.

## Background

The prevalence of allergy-related diseases, such as asthma, allergic rhinoconjunctivitis (ARC) and eczema, increased substantially in the second half of the twentieth century. Together, these diseases are the most common noncommunicable diseases among children worldwide [1]. Whilst the prevalence varies widely between counties, we tend to find higher rates of these diseases in developed countries. In Europe, the reported cumulative incidence of asthma, ARC and eczema at 8 years of age is around 15, 16 and 34%, respectively [2]. These allergy-related diseases often appear to share some common pathophysiological pathways, and children who are also sensitised to common allergens are at a greater risk of other allergy-related diseases [2, 3]. Both genetic and environmental factors are thought to contribute to the complex aetiology of these diseases [4].

The influence of the duration and exclusivity of breastfeeding on allergy-related diseases has been widely debated for decades. While the primary role of breast milk is to nourish and hydrate newborn infants, it also provides a direct immune defence against pathogens, decreasing the risk of respiratory and gastrointestinal infections in infants [5].

Whilst many studies have reported a protective effect of breastfeeding on allergy-related diseases, others have found that it may even increase the risk of these diseases. Overall, two recent meta-analyses found a reduced risk of the composite outcome asthma/wheeze with longer breastfeeding [6, 7], a possible reduction in the risk of ARC [7] and no apparent effect on the risk of eczema [6, 7]. As highlighted by these meta-analyses, the conflicting results of the original studies are likely due to a combination of the methodological challenges in breastfeeding research, and further complicated by the variations in how breastfeeding exposure and the allergy-related outcomes are defined. In likeness with many observational studies, the internal validity of research into breastfeeding is threatened by the risk of reverse causality, recall bias of breastfeeding exposure and incomplete adjustment for confounding factors [7]. The systematic review and meta-analysis by Garcia-Larsen et al. further emphasised the problem with publication bias, and estimated that the lack of control for confounding alone accounted for a high risk of bias in 30–35% of all included studies [6]. Additionally, there is heterogeneity in definitions used for the allergy-related diseases, and the stringency of these definitions may influence the apparent effect of breastfeeding. For example, the definition of asthma varies widely from parental reported wheeze to an asthma diagnosis after a physician examination [6–11].

More recently, the impact of age of introduction to complementary foods on allergy-related diseases has also been debated. Randomised controlled trials have produced some promising results for early introduction of peanut and egg in high risk infants to prevent food allergy to these specific allergens [12, 13]. However, it is uncertain if the timing of introduction to complementary foods, in general, influences the development of allergy-related disease. This was investigated in a recent systematic review by Obbagy et al. [14], which concluded that there is no certain relationship between the age of complementary food introduction and asthma, eczema or food allergies. They had insufficient studies to assess the relationship for ARC. This systematic review also emphasised that many of the included studies suffered from the same methodological challenges faced by studies investigating the effect of breastfeeding [6, 11, 14]. As such, a larger prospective cohort study that attempts to address these limitations is warranted for both breastfeeding and age of complementary food introduction.

In the current study, we have used data from the Prevention of Allergy among Children in Trondheim (PACT) study. Our aim was to examine the associations between the duration of breastfeeding, the age of introduction to complementary foods, and the allergy related outcomes, asthma, wheeze, ARC and eczema, at two and six years of age.

## Methods

### Prevention of allergy among children in Trondheim study

This is a substudy of the PACT study and is based on all children for whom we have data on breastfeeding duration or complementary food introduction and allergy-related disease at two or six years. The main PACT study was a controlled, prospective cohort study of pregnant women and their children in primary healthcare in Trondheim, Norway. The PACT study aimed to reduce the incidence of allergy-related diseases among children through lifestyle recommendations given to pregnant women and families of young children [15]. The recommendations included in the intervention were to reduce tobacco exposure, reduce housing dampness and mould, and to increase maternal and infant intake of fatty fish and cod liver oil. The introduction of fish was recommended as dinner and sandwich spread from when the child was six months of age [16]. After the intervention commenced, these recommendations were given to all expecting families, as a public health strategy, regardless of whether they were participating in the study or not. No aspect of the intervention was aimed at breastfeeding, formula feeding or the timing of complementary foods introduction [15].

Throughout the PACT study, the national recommendations in Norway were exclusive breastfeeding for the first six months of life, with introduction of complementary foods at six months together with continued breastfeeding up to one year of age [16]. This is in line with the current recommendations from the World Health Organization, except that their advice includes continued breastfeeding up to two years of age or beyond [17].

### Study design and participants

**Fig. 1:**
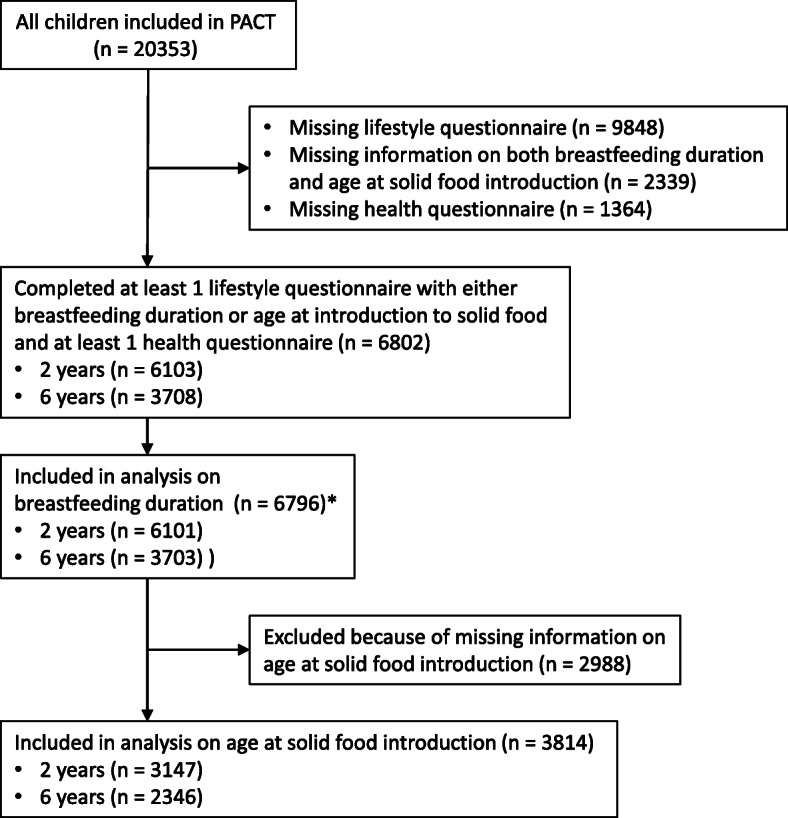
Flow chart of participants

Participating families were recruited at routine prenatal and child health follow-ups at general practices and community health centres throughout Trondheim [15]. All seven community-based midwives, all 20 maternity health centres, and 32 of 35 general practices in Trondheim participated. Socio-demographic characteristics and relevant risk factors were obtained from up to four lifestyle questionnaires completed during pregnancy and when the child was six weeks, one year and two years old. In addition, child health questionnaires, focusing primarily on signs and symptoms of allergy-related disease, were completed when the child was two and six years old. Inclusion to the PACT study commenced in 2000 with recruitment of pregnant women, as well as 6-week, 1-year, 2-year and 6-year-old children. The inclusion period for pregnant women ended in 2006 and the last 6-year-old questionnaires were completed in 2014. During the inclusion period there were approximately 2150 deliveries per year in Trondheim [18], however we do not have accurate information about how many families chose not to participate in the PACT study. The questions regarding allergy-related diseases were adapted from the International Study of Asthma and Allergies in Childhood (ISAAC) questionnaire with adjustment made to suit the age group and were previously assessed in a reliability study conducted by the PACT research group [19]. The current study consists of all children whose family had completed at least one lifestyle questionnaire, including information on breastfeeding or introduction of first complementary food, and at least one child health questionnaire (Fig. 1).

### Outcome variables

The studied outcomes were asthma, wheeze, ARC and eczema in children at two and six years of age. While asthma and ARC are considered as more defined diseases at six years, the peak prevalence of eczema occurs at a younger age (18–21). Therefore, a cumulative prevalence of eczema at six years of age was calculated to assess the effect of breastfeeding and complementary food on the risk of eczema at any time up to six years. The outcomes were defined according to the answers in the child health questionnaires (Table 1). To be able to compare these results to studies with different outcomes definitions, this study included both the parental reported doctor-diagnosed asthma, and parental reports of symptoms of wheeze.

**Table 1:** Outcome variables as defined based on questionnaires

Allergy-related condition	Time point(s)	Questions used to define cases
Asthma (ever)	2 years	“Has the child ever been diagnosed with asthma by a doctor”
Asthma (current)	6 years	As asthma (ever) AND “Has the child been treated with tablets, inhalation medications or other treatments for wheezing, tightness in the chest or asthma during the last 12 months?
Wheeze (ever)	2 years	“Has the child ever had whistling in the chest?” AND “Has the child ever had episodes of wheezing or tightness in the chest?
Wheeze (current)	6 years	As for wheeze (ever) AND “Has the child had whistling, rattling or tightness in the chest in the past 12 months?
ARC (ever)	2 & 6 years	“Has the child ever had hay fever, sneezing or itchy-watery eyes?
Eczema (ever)	2 & 6 years	“Has the child ever had eczema?” AND “Has the child ever had an itchy rash which came and went over at least six months?
Eczema (current)	6 years	As for eczema (ever) AND “Has the child during the last 12 months used medications, balms, creams, tablets or herbal medicines for eczema?

The age of onset of the different outcomes was also recorded in order to account for reverse causality, except for wheeze where the questionnaires did not provide such data.

### Exposure variables

The aim of this study was firstly to investigate whether the duration of breastfeeding had any effect on the development of asthma, wheeze, ARC or eczema. We defined breastfeeding as any breastfeeding, regardless of whether the child had been introduced to any solids or formula. A cut-off at six months divided the participants into two groups: those who had been breastfed six months or more, and those breastfed less than six months. This information was collected from the lifestyle questionnaires completed at six weeks, one year and two years. At each of these time points, participating families were asked if the child ever had received breast milk and if they had been weaned and at what age. The information on breastfeeding duration was collected from the closest available questionnaire, with respect to age of weaning, and resulted in a maximum recall from six weeks to two years.

Subsequently, we studied the risk of developing allergy-related diseases with respect to age at introduction of complementary foods. Participating families were asked about the age at which the child had been introduced to porridge (rice, corn and wheat), boiled and raw vegetables, fruits, commercial or homemade dinners, fish, cow’s milk, and egg. The time of introduction to complementary foods was defined as the earliest age (in months) the child had started with any of the mentioned dietary options. Based on this age, the children were categorised into two groups: those introduced at six months of age or later, and those introduced before six months of age. This information was obtained from the lifestyle questionnaire completed when the child was one year old and resulted in a maximum recall of one year. Finally, we analysed if a period of overlap between breastfeeding and nourishment with complementary foods had any effect on the development of allergy-related diseases. A minimum of two months overlap was used to distinguish the two groups.

### Statistics

All statistical analyses were conducted using Stata/MP 15 (StataCorp, College Station, Texas). Univariable logistic regression was used to obtain crude odds ratios (ORs) for each studied exposure and outcome. Potential confounders were identified a priori and examined in multivariable analyses before determining a final model and the results are presented as adjusted odds ratios (aORs). Statistical significance was assessed using 95% CIs. In sensitivity analyses, we considered the potential effects of recall bias and reverse causality on our results and examined if the relationship between longer breastfeeding duration and allergy-related diseases may be modified by the introduction of formula before six months. First, the breastfeeding analyses were repeated in the subgroup of families with maximum of one-year recall for age of weaning. Second, all analyses were repeated after exclusion of children reported to have developed symptoms of any of the allergy-related diseases before six months of age. Third, we separated the group which reported any breastfeeding for at least six months into those who had or had not introduced infant formula before six months of age and compared each of these subgroups to children who were weaned before six months.

In all analyses, we considered the following covariates as possible confounders: gender, maternal age, older siblings, first degree relative with allergy, maternal smoking during the child’s first two years, birthweight, and socio-economic status (SES). Ultimately, the mean income of the family’s postcode of residence presence was used as an indicator of SES and the presence of older siblings was excluded from the final models as described below. Information regarding covariates were collected from the lifestyle questionnaires, except for mean income, which was gathered from the Norwegian assessment roll from 2009 [20].

There is no single best indicator of SES [21] and we found the average income in the postcode of residence to be the most appropriate alternative in the current study. Other indicators of SES collected in the PACT study were homeownership and parental level of education. The former provided poor discrimination of SES due to the high proportion of homeownership in Trondheim, and the latter was subject to a large proportion of missing data because information on parental level of education was not collected throughout the entire study period. Before using the average income of the postcode of residence in our analyses, we conducted a number of tests to examine its suitability. We confirmed that it corresponded well with other variables commonly associated with SES, such as parental smoking behaviour, maternal age and homeownership (Suppl. Table 1, Additional File 1). Since parental education did not demonstrate a clear association with the average income of postcode of residence, we investigated the effect of including either or both variables as covariates in our analyses. In order to do this we ran parallel logistic regression models and confirmed that the estimated associations between breastfeeding or introduction to complementary food and the allergy-related diseases were near identical regardless of whether we adjusted for average postcode income or maternal education level in the subgroup of participants with information on both SES indicators (*n* = 1669 to 2156 depending on which allergy-related outcome data not shown). In order to maintain a parsimonious model, the following variables were not included as covariates in the final models after preliminary analyses: the presence of older siblings, exposure to paternal smoking and household pets in the first year of life, and day-care attendance before one year of age. The presence of older siblings was not excluded because it did not significantly affect the results when included as a covariate and we did not have this information for a high proportion of the children. Since parental smoking was highly correlated with maternal smoking, we opted to include only the latter. Exposure to household pets was excluded because it was considered unlikely to be a substantial confounding factor and we observed that it did not significantly alter the regression results when included as a covariate. Similarly, we saw little indication that day-care attendance before one-year was associated with breastfeeding or introduction to complementary foods at six months in our population and this was not included as a covariate in the models. Respiratory tract infections and antibiotic use were considered potential mediators of the effect of breastfeeding on allergy-related diseases and not included in the regression models.

## Results

The PACT study includes over 20,000 children, however nearly half of these families were recruited when the children were 6-years-old such that for 9848 children we had no information from the lifestyle questionnaires due to the design of the study. In total, 6802 children could be included in this current study, of which 6103 and 3708 had completed the child health questionnaire at two and six years, respectively (Fig. 1). In addition to those who were missing the lifestyle questionnaires, 2339 and 1364 could not be included due to missing information on the exposures or outcomes, respectively. We do not have further information on reasons for non-participation or loss to follow up. However, a previous non-participant study showed a tendency of lower prevalence of parental smoking and proportion of with family history of allergy-related disease in the PACT population, but none of these differences were statistically significant [15].

The mean age of the children at the two follow up points were 2.5 years (range 1.7–3.0 years) and 6.4 years (range 5.6–7.5 years). The prevalence of each of the studied allergy-related diseases at two and six years are presented in Table 2. Characteristics of families included in analyses of breastfeeding duration and timing of complementary food introduction are presented in the relevant sections, below. Families excluded from the current analyses due to missing child health questionnaire data reported a shorter breastfeeding duration and a higher percentage of formula supplemented children compared to those included in the current study. In this group, there was a lower proportion with older siblings, furry pets, antibiotic treatment within the first year of life, and reported family history of allergy-related disease (Supplementary Table S2, Additional File 1).

**Table 2:** Prevalence of allergy-related diseases

Disease and age	N^*^	***n***	%	95% CI
Asthma				
**2 years (ever)**	6101	403	6.6	6.0, 7.3
**6 years (current)**	3705	175	4.7	4.1, 5.5
Wheeze				
**2 years (ever)**	5922	1577	26.6	25.5, 27.8
**6 years (current)**	3668	388	10.6	9.6, 11.6
ARC				
**2 years (ever)**	5562	354	6.4	5.8, 7.0
**6 years (ever)**	3438	424	12.3	11.3, 13.5
Eczema				
**2 years (ever)**	5927	998	16.8	15.9, 17.8
**6 years (current)**	3648	505	13.8	12.8, 15.0
**6 years (ever)**	3630	710	19.6	18.3, 20.9

^*^Children with available information on duration of breastfeeding or age at introduction to complementary foods are included. The numbers vary because of missing outcome data

### Breastfeeding duration

For the 6796 children with available information on breastfeeding, the mean duration of breastfeeding was 11 months (SD 5.6) and 5670 (84%) had been breastfed for six months or more. A longer duration of breastfeeding was positively associated with older mothers, and parents with higher education and who lived in areas with higher mean income (Table 3). The duration of breastfeeding was also strongly correlated to parents smoking habits, and children who were breastfed longer were more likely to have non-smoking parents. Additionally, they were less likely to have been born with low birthweight.

**Table 3:** Characteristics of the participants stratified according to duration of breastfeeding

	Duration of any breastfeeding (***N*** = 6796)			
	≥ 6 months (***n*** = 5695)		< 6 months (***n*** = 1101)	
Characteristics	***n***^**a**^		***n***^**a**^	
Maternal age at birth, years, mean (SD)	5670	30.2 (4.4) range: 16.8–44.5	1095	28.7 (5.1) range: 17.0–44.2
Maternal education, *n* (%)	2625		392	
< 12 years (less than high school)		116 (4.4)		62 (15.8)
12–16 years (up to 4 years university)		1298 (49.5)		237 (60.5)
> 16 years (more than 4 years university)		1211 (46.1)		93 (23.7)
Mean income^b^, NOK, mean (SD)	5465	255,414 (29175)	1053	250,191 (30405)
Family history of allergy^c^, *n* (%)	5675	4200 (74.0)	1092	784 (71.8)
Older sibling, *n* (%)	3943	2245 (56.9)	731	401 (54.9)
Maternal smoking, *n* (%)				
During pregnancy	2020	92 (4.6)	316	55 (17.4)
During child’s first 2 years	5638	887 (15.7)	1078	391 (36.3)
Paternal smoking, *n* (%)	5397	1072 (19.9)	994	330 (33.2)
Child exposed to smoke during first 2 years^d^, *n* (%)	5675	1462 (25.8)	1095	502 (45.8)
Pets^e^, *n* (%)	5695	1143 (20.1)	1101	264 (24.0)
Birthweight, gram, mean (SD)	5625	3608 (552) range: 640–5560	1072	3463 (705) range: 585–5960
Birthweight < 2500 g, *n* (%)	5625	156 (2.8)	1072	88 (8.2)
Sex, male*, n* (%)	5693	2798 (49.2)	1101	581 (52.8)
Lower respiratory tract infection within first year^f^, *n* (%)	3217	333 (10.4)	606	78 (12.9)
Antibiotic treatment within first year, *n* (%)	3746	756 (20.2)	695	163 (23.5)
Introduced to formula < 6 months of age, *n* (%)	5625	966 (17.2)	1093	972 (88.9)^g^
Start of day-care < 12 months of age, *n* (%)	5051	394 (7.8)	930	65 (7.0)

^a^:The numbers vary because of missing data

^b^: Yearly income in Norwegian krone based on postcode (range: 174967 to 319,333 for both groups)

^c^: Mother, father or common child with parental reported asthma, ARC or eczema

^d^: Parental or indoor smoking; ^e^: Reported cat, dog or other furry pets

^f^: Pneumonia or bronchitis

^g^: questionnaire data indicates children started with formula after 6 months of age despite having been weaned before 6 months

As would be expected, we also observed that children who were breastfed for more than six months tended to receive less infant formula (Table 3). In the subgroup of infants with information on both breastfeeding duration and complementary food introduction age (*n* = 3808), we found that only 993 (26%) of families waited until six months before introducing complementary foods, regardless of breastfeeding duration. However, those families who breastfed for less than six months was substantially less likely to delay complementary food introduction until six or later months with only 60 of 606 (10%) families falling into this category (Suppl. Table S3, Additional File 1).

### Breastfeeding and allergy-related disease

When assessing the relationship between breastfeeding for at least six months and allergy-related disease, the crude ORs suggested a statistical association between longer breastfeeding and a lower cumulative incidence of both asthma and wheeze at two years, as well as a lower prevalence of current wheeze at six years of age (Table 4). After adjusting for possible confounding, longer breastfeeding duration was still associated with lower cumulative incidence of asthma and wheeze at two years although the associations were weaker and no longer statistically significant: aOR 0.77 (95% CI 0.59, 1.02) and aOR 0.90 (95% CI 0.53, 1.07), respectively. On the other hand, there remained a strong and statistically significant reduction in current wheeze at six years associated with longer breastfeeding (aOR 0.71, 95% CI 0.53, 0.94). In absolute terms, we observed a 3.4% lower prevalence of wheeze at six years among children who had been breastfed for at least six months compared to those who were breastfed for a shorter period (10.1% vs 13.5%) (Table 4). A strong and statistically significant reduction in crude OR was also observed for ARC at two years, which persisted after adjustment for possible confounders (aOR 0.65, 95% CI 0.49, 0.86) and after exclusion of symptomatic children before six months of age (Suppl. Table S4, Additional File 1). A trend for a continued protective effect of longer breastfeeding was also seen for ARC at six years (aOR 0.77, 95% CI 0.58, 1.04). For eczema and two and six years, although observed that longer breastfeeding duration was associated with slight increased odds of eczema in both the crude and adjusted analyses, these estimates were associated with wide confidence intervals and were not statistically significant (Table 4). When excluding children with symptoms of allergy-related disease before six months of age, the analyses showed similar trends for all outcomes, but with broader confidential intervals due to fewer eligible children (Suppl. Table S4, Additional File 1). Similarly, we saw approximately the same results in the analyses which excluded participants with more than one-year recall of breastfeeding (Suppl. Table S5, Additional File 1).

**Table 4:** Relation between breastfeeding duration and allergy-related diseases

	Duration of any breastfeeding (***N*** = 6796)				Odds ratio^**a**^ (95% CI)	
	≥ 6 months (***n*** = 5695)		< 6 months (***n*** = 1101)			
**Disease and age**	***n***^**b**^	***n*** **(%)**	***n***^**b**^	***n*** **(%)**	**Crude**	**Adjusted**^**c**^
Asthma						
**2 years (ever)**	5135	319 (6.2)	964	83 (8.6)	0.70 (0.55, 0.90)	0.77 (0.59, 1.02)
**6 years (current)**	3134	142 (4.5)	566	32 (5.7)	0.79 (0.53, 1.17)	0.79 (0.51, 1.21)
Wheeze						
**2 years (ever)**	4981	1288 (25.9)	939	288 (30.7)	0.79 (0.68, 0.92)	0.90 (0.76, 1.07)
**6 years (current)**	3098	312 (10.1)	565	76 (13.5)	0.72 (0.55, 0.94)	0.71 (0.53, 0.95)
ARC						
**2 years (ever)**	4690	268 (5.7)	871	86 (9.9)	0.55 (0.43, 0.71)	0.65 (0.49, 0.86)
**6 years (ever)**	2913	348 (12.0)	520	76 (14.6)	0.79 (0.61, 1.04)	0.77 (0.58, 1.04)
Eczema						
**2 years (ever)**	4994	853 (17.1)	931	145 (15.6)	1.12 (0.92, 1.35)	1.16 (0.94, 1.44)
**6 years (current)**	3087	436 (14.1)	556	69 (12.4)	1.16 (0.88, 1.52)	1.15 (0.86, 1.55)
**6 years (ever)**	3071	613 (20.0)	554	97 (17.5)	1.17 (0.93, 1.49)	1.18 (0.92, 1.53)

^a^: For duration of any breastfeeding for six months or more versus less than six months

^b^: The numbers vary because of missing data

^c^: Adjusted for sex, maternal age, mean income, first degree relative with allergy, maternal smoking during the child’s first two years and birthweight

We also investigated the potential modifying effect of early introduction to infant formula by dividing the exposure status of children into three categories: those who were breastfed for less than six months, those who were breastfed for six months but also received infant formula before six months, and those who were breastfed for at least six months and did not receive infant formula during these first six months. For asthma, wheeze and eczema, we found that the risk of these allergy-related diseases was approximately the same among children breastfed for at least six months, regardless of whether they had been introduced to infant formula before six months of age (Suppl. Table S6, Additional File 1). On the other hand, the reduction in ARC observed among children with a longer duration of breastfeeding appears to be predominantly present in the subgroup that did not receive infant formula before six months of age. Specifically, the risk of ARC at two years of age was reduced in those breastfed without any infant formula up to six months (aOR 0.61, 95% CI 0.46, 0.82) compared to those who were weaned before six months. Although a reduced risk was also seen for children who received supplementation with formula before six month and ongoing breastfeeding, the association was weaker and the estimate was less certain (aOR 0.78, 95% CI 0.54, 1.15). Among six year olds, we observed a reduced risk of ARC for those who were breastfed without the introduction of formula up to at least six months (aOR 0.74, 95% CI 0.55, 1.01), however the risk of ARC among children who had received formula before six months was approximately equal to those who were weaned before six months (aOR 1.04, 95% CI 0.71, 1.51).

### Timing of introduction to complementary foods and allergy-related disease

Our data included information on the age of complementary food introduction for 3814 children. Among these infants, the mean age of introduction was 4.6 months (SD 1.3) and 26% were introduced to complementary foods at the age of six months or older. Later introduction was positively correlated to higher maternal education and non-smoking parents (Table 5). The group that was introduced to complementary foods at six months or older was less likely to have received milk formula early in life. In this group, 933 of 993 (94%) had been breastfed six months or more compared to 2269 of 2815 (81%) in the early introduced group (Suppl. Table S3, Additional File 1).

**Table 5:** Characteristics of the participants stratified according to age at introduction of complementary foods

	Age at introduction of complementary foods (***N*** = 3814)			
	≥ 6 months (***n*** = 996)		< 6 months (***n*** = 2818)	
**Characteristics**	***n***^**a**^		***n***^**a**^	
Maternal age at birth, years, mean (SD)	994	31.2 (4.2) range: 18.0–43.8	2810	29.6 (4.5) range: 17.0–43.7
Maternal education, *n* (%)	596		1409	
< 12 years (less than high school)		17 (2.9)		106 (7.5)
12–16 years (up to 4 years university)		288 (48.3)		749 (53.2)
> 16 years (more than 4 years university)		291 (48.8)		554 (39.3)
Mean income^b^, NOK, mean (SD, range)	952	256,368 (28674)	2699	254,101 (29284)
Family history of allergy^c^, *n* (%)	995	768 (77.2)	2812	2147 (76.4)
Older sibling, *n* (%)	995	588 (59.1)	2809	1570 (55.9)
Maternal smoking, *n* (%)				
During pregnancy	538	10 (1.9)	1126	91 (8.1)
During child’s first 2 years	994	107 (10.8)	2807	597 (21.3)
Paternal smoking, *n* (%)	981	170 (17.3)	2717	680 (25.0)
Child exposed to smoke during first 2 years^d^, *n* (%)	995	201 (20.2)	2817	923 (32.8)
Pets^e^, *n* (%)	996	239 (24.0)	2818	633 (22.5)
Birthweight, gram, mean (SD, range)	992	3583 (576) range: 1100–5500	2808	3587 (577) range: 585–5370
Birthweight < 2500 g, *n* (%)	992	31 (3.1)	2808	106 (3.8)
Sex, male, *n* (%)	996	465 (46.7)	2818	1418 (50.3)
Lower respiratory tract infection within first year^f^, *n* (%)	992	101 (10.2)	2809	307 (10.1)
Antibiotic treatment within first year, *n* (%)	995	203 (20.4)	2812	689 (24.5)
Introduced to formula < 6 months of age, *n* (%)	992	127 (12.8)	2810	897 (31.9)
Start of day-care < 12 months of age, *n* (%)	806	70 (8.7)	2281	200 (8.8)

^a^: The numbers vary because of missing data

^b^: Yearly income in Norwegian krone based on postcode (range: 174967 to 319,333 for both groups)

^c^: Mother, father or common child with parental reported asthma, ARC or Eczema

^d^: Parental or indoor smoking

^e^: Reported cat, dog or other furry pets

^f^: Pneumonia or bronchitis

Whilst, we found that late complementary food introduction (six months or older) was associated with a lower risk of wheeze at two years in the crude analyses, this association was weaker and no longer statistically significant in the adjusted model (aOR 0.85, 95% CI 0.70, 1.05) (Table 6). Otherwise our investigations did not demonstrate any convincing associations between age of introduction to complementary foods and allergy-related diseases (Table 6 and Suppl. Table S7, Additional File 1).

**Table 6:** Relation between age at introduction to complementary foods and allergy-related diseases

	Introduction to complementary foods (***N*** = 3814)				Odds ratio^**a**^ (95% CI)	
	≥ 6 months (***n*** = 996)		< 6 months (***n*** = 2818)			
**Disease and age**	***n***^**b**^	***n*** **(%)**	***n***^**b**^	***n*** **(%)**	**Crude**	**Adjusted**^**c**^
Asthma						
**2 years (ever)**	818	46 (5.6)	2328	143 (6.1)	0.91 (0.65, 1.28)	0.92 (0.64, 1.34)
**6 years (current)**	644	30 (4.7)	1699	75 (4.4)	1.06 (0.69, 1.63)	1.18 (0.75, 1.86)
Wheeze						
**2 years (ever)**	797	173 (21.7)	2256	582 (25.8)	0.80 (0.66, 0.97)	0.85 (0.70, 1.05)
**6 years (current)**	637	70 (11.0)	1683	173 (10.3)	1.08 (0.80, 1.45)	1.13 (0.83, 1.54)
ARC						
**2 years (ever)**	757	36 (4.8)	2127	133 (6.3)	0.75 (0.51, 1.09)	0.88 (0.60, 1.30)
**6 years (ever)**	603	76 (12.6)	1591	203 (12.8)	0.99 (0.74, 1.31)	1.09 (0.81, 1.47)
Eczema						
**2 years (ever)**	793	118 (14.9)	2267	360 (15.9)	0.93 (0.74, 1.16)	0.98 (0.77, 1.24)
**6 years (current)**	631	82 (13.0)	1670	224 (13.4)	0.96 (0.74, 1.26)	0.96 (0.72, 1.28)
**6 years (ever)**	629	113 (18.0)	1661	328 (19.8)	0.89 (0.70, 1.13)	0.89 (0.69, 1.13)

^a^: For introduction to complementary foods at six months or older versus younger than six months

^b^: The numbers vary because of missing data

^c^: Adjusted for sex, maternal age, mean income, first degree relative with allergy, maternal smoking during the child’s first two years and birthweight

Finally, we considered the influence of an overlapping period of breastfeeding and introduction to complementary foods on allergy-related disease development. On average, the children in the PACT study were breastfed for 6.2 months (SD 4.7) after the introduction of complementary foods and 2837 of 3485 (82%) had a minimum of two months overlap. We found that the development of allergy-related disease was not convincingly associated with this overlap between breastfeeding and complementary food introduction (Suppl. Table S8, Additional File 1).

## Discussion

### Main results

Using prospective data from the PACT study, we found that a high proportion of women in central Norway continued breastfeeding beyond six months of age. However, the majority of families reported that they started introducing complementary foods before six months of age. Upon examining the relationship between these factors and allergy-related disease, we found that a longer duration of any breastfeeding is not conclusively associated with parental reported doctor-diagnosed asthma but may reduce the risk of wheeze at six years. We also observed an apparent protective effect of breastfeeding for at least six month and ARC at two years of age. At six years of age, we saw an ongoing tendency to reduced ARC among infants breastfed over six months. However, breastfeeding duration was not associated with eczema in our data, and neither the timing of introduction to complementary foods nor overlap between breastfeeding and complementary food introduction demonstrate any clear relation to asthma, wheeze, ARC or eczema.

Our results for asthma at six years are consistent with the findings of another large Norwegian cohort. Using data from the Norwegian Mother and Child Cohort Study (MoBa), Lossius et al. found that breastfeeding over one year did not appear to impart a protective effect against asthma compared to shorter durations [10]. Although they adopted a more stringent definition of asthma, their prevalence of 4.7% is comparable to our estimated prevalence of doctor diagnosed asthma. The findings of The Promotion of Breastfeeding Intervention Trial, the only cluster randomised trial on breastfeeding and asthma, also supports our results as they concluded that longer breastfeeding does not reduce asthma diagnosis at 6.5 years [8].

In contrast to these findings for asthma, both recent meta-analyses found a reduced risk of asthma/wheeze [7] and recurrent wheeze [6] with a longer duration of total breastfeeding. A summary of the results from these meta-analyses for asthma/wheeze and the other allergy-related diseases can be found in Table 7. Both meta-studies raised the suspicion of overstatement of the protective effects of breastfeeding on asthma/wheeze. Garcia-Larsen et al. [6] described their conclusion as having a very low grade of evidence. This was primarily due to failure to adjust for confounders, but also because of evidence of publication bias with many small studies reporting protective effects. Correspondingly, Lodge et al. [7] described weaker protective estimates in studies of higher methodological quality, particularly with respect to recall bias, but also in terms of controlling for confounders and the number of participants. Indeed, they found no association between breastfeeding and reduced asthma/wheeze when including only cohort studies. Whilst the risk of publication bias is evident, these meta-analyses of asthma and wheeze as a composite outcome, together with our own results, indicate that a longer duration of breastfeeding may have a prolonged protective effect primarily on symptomatic wheezing rather than doctor-diagnosed asthma. This may be due to both ongoing effects of early protection against respiratory infections as well as possible protection against infection also after weaning [9, 22]. The results from the Millennium Cohort Study, United Kingdom, also demonstrate a clear dose-response effect of breastfeeding on early transient wheeze phenotype, but less certain relationships with late onset or persistent wheezing phenotypes [23].

**Table 7:** Association between breastfeeding duration and allergy-related disease in recent meta-analyses [6, 7]

Condition & age	Comparison for breastfeeding duration^**a**^	All studies included		Prospective cohort studies		Ref
		***N***^**b**^	OR	***N***	OR	
**Wheeze**						
0–4 yo	TBF > 5-7mo vs. < 5-7mo	9	0.90 (0.72, 1.12)	6	0.89 (0.65, 1.21)	[6]
5–14 yo	TBF > 5-7mo vs. < 5-7mo	2	0.86 (0.70, 1.06)	1	0.88 (0.71, 1.09)	[6]
**Recurrent wheeze**						
0–4 yo	TBF > 5-7mo vs. < 5-7mo	6	0.79 (0.61, 1.04)	3	0.73 (0.56, 0.96)	[6]
5–14 yo	TBF > 5-7mo vs. < 5-7mo	9	0.76 (0.62, 0.92)	4	0.82 (0.66, 1.03)	[6]
**Asthma**^**c**^						
5–18 yo	More vs. Less	29	0.90 (0.84, 0.97)	13	0.94 (0.80, 1.11)	[7]
**Allergic rhinitis / rhinoconjunctivitis**						
0–4 yo	TBF > 5-7mo vs. < 5-7mo	2	0.81 (0.64, 1.02)	--- ^d^		[6]
0–5 yo	More vs. Less	6	0.79 (0.63, 0.98)	2	0.91 (0.74, 1.13)	[7]
5–14 yo	TBF > 5-7mo vs. < 5-7mo	1	1.14 (0.75, 1.72)	--- ^d^		[6]
5–18 yo	More vs. Less	9	1.05 (0.99, 1.12)	4	1.12 (1.02, 1.24)	[7]
**Ezcema**						
0–2 yo	More vs. Less	16	0.95 (0.85, 1.07)	15	0.97 (0.86, 1.10)	[7]
0–4 yo	TBF > 5-7mo vs. < 5-7mo	4	0.93 (0.80, 1.07)	3	0.97 (0.75, 1.12)	[6]
2–18 yo	More vs. Less	20	1.09 (0.99, 1.20)	6	1.36 (0.90, 2.06)	[7]
5–14 yo	TBF > 5-7mo vs. < 5-7mo	2	1.35 (1.07, 1.72)	--- ^d^		[6]

*TBF* Total (any) breastfeeding

^a^Both meta-analyses report several comparisons based of different definitions of breastfeeding duration and exclusivity, here summarise the results from the comparison which would have included our own categorisation of breastfeeding duration. Meta-analysis by Lodge et al. [7] included a wide variety of studies comparing “more” vs “less” breastfeeding, whilst Garcia-Larsen et al. [6] completed separate meta-analyses for more specific comparisons of breastfeeding duration

^b^Number of studies in the meta-analysis

^c^Lodge et al. include parental reports of wheeze as “asthma” in their meta-analysis

^d^Only prospective cohort studies available for inclusion in these analyses

In our analyses, we also found that breastfeeding with a duration over six months tended to reduce the risk of ARC until school age. Lodge et al. found a similar risk reduction in their meta-analysis, but rightly pointed out that it is difficult to differentiate between viral and allergic rhinitis in young infants, such that the apparent protective effect of breastfeeding may be primarily against infectious rather than allergic rhinitis [7]. The lack of association between breastfeeding duration and eczema observed in our study is also consistent with the results from the meta-analyses by Lodge et al. and Garcia-Larsen et al. [6, 7]. Furthermore, we found no statistically significant effects of age at introduction to complementary foods or continued breastfeeding while these foods were being introduced. This strengthens the conclusion from the meta-analyses by Garcia-Larsen et al. and Obaggy et al. which found no relation between age of introduction and allergy-related diseases [6].

### Strengths of the study

The strengths of this study are its size, prospective design and comprehensive questionnaires. With information on approximately 6000 two-year-old and 3700 six-year-old children, our study represents one of the larger prospective cohort studies assessing the association between breastfeeding and eczema and ARC. Although there have been some larger cohort studies considering the influence of breastfeeding on asthma or wheeze, we also consider it a strength that all of these conditions have been examined together. In particular, the inclusion of both wheeze and doctor-diagnosed asthma allows us to examine the impact of the stringency of these definitions on the apparent association between breastfeeding and allergy-related disease. Furthermore, the prospective design minimises the risk of recall bias and allow us to consider the potential effect of reverse causality. In sensitivity analyses we considered both of these potential sources of errors, and found that our results were largely unaffected by the exclusion of participating families who reported weaning more than one year ago and when excluding children with symptom debut of any allergy-related disease before six months of age. With respect to recall bias, there is also evidence that recall of breastfeeding duration among Norwegian women is highly accurate even up to 20 years later [24].

Another strength of the PACT study is the detailed information found in the lifestyle questionnaires allowing us to control for potential confounding factors. As identified in the recent meta-analyses, incomplete adjustment for key confounders has been a major limitation of studies assessing the association between duration and exclusivity of breastfeeding, timing of complementary food introduction and the development of allergy-related diseases [6, 7]. The confounding effect of family history of allergic disease and socio-economic status (SES) are considered particularly important in this context, both of which were included in our multivariable analyses together with other potential confounding factors. Family history of allergic disease in a first degree relative was included as covariate based on parent reports. Interestingly, we noted that family history was not significantly associated with either duration of breastfeeding or the age of introduction to complementary foods in our dataset. The challenges of controlling for SES are discussed in more detail in the limitations section, below. We also note that earlier studies have considered lower respiratory tract infections and antibiotic use within first year of life as possible confounders [7, 25, 26]. These variables have not been included as covariates in our analyses as we regard these to be potential mediators of the effect of breastfeeding on allergy-related disease. Adjustment for such mediating variables can bias the total effect estimates and should be avoided.

Previous studies have been criticised for evaluating allergic outcomes before breastfeeding termination and too early in childhood, when allergy-related disorders are harder to distinguish from infectious diseases [27]. By two years, almost all the children in the current study were weaned from breast milk and a persisting effect should be possible to detect. A diagnosis of asthma before five years may be misclassified infant wheezing and thus not an estimate of a true atopic condition. By examining the relations further at six years of age, our study allows for analyses of the time aspect in addition to account for the possible false diagnostics at two years.

### Limitations of the study

The limitations to our study are the possibility of misclassification bias due to parental reported outcomes and residual confounding due to missing information or misclassification of potential confounders. There is also a risk of selection bias and a limited generalizability outside of Scandinavia. The parental reported outcomes are subjected to some degree of recall bias and are not as rigorous as physician examination or more objective measurements, like spirometry. In particular, our definitions of ARC and wheeze may identify infectious conditions rather than allergic ones. On the other hand, the reliability study on the questionnaires in PACT showed that the questions used for asthma and eczema underestimated, rather than overestimated, the prevalence of these conditions compared to the children’s medical records [19]. Future studies should also consider using the Norwegian Prescription Database (NorPD), primarily for confirming the diagnosis of asthma. This was not possible for the current analysis since only 15% (*n* = 1022) of the included children were born after the establishment of this database in 2004. Reassuringly, we did not find evidence for recall bias upon excluding children with a recall period longer than one year. Residual confounding is another source of bias which we cannot exclude. Although our study accounts for many of the identified confounders, the PACT study did not include information on mode of delivery and mothers’ body mass index. Caesarean delivery has been previously associated with both breastfeeding duration [28] and allergy-related diseases [29]. Although there is some evidence that elective Caesarean delivery is particularly associated with lower rates and duration of breastfeeding [30], it is unclear if this association represents a causal biological effect or is confounded by factors such as intending to breastfeed which has been found to be lower among those who elect to deliver via Caesarean section [28]. Whilst there may be residual confounding because we have not taken mode of delivery into account, the national Caesarean section rate during the study period ranged from around 14 to 17% and approximately two thirds of these were emergency Caesarean sections [31]. Gestational age at birth is another such potential confounder that could not be included in the current analyses due to a high proportion of missing information. Nonetheless, we do not believe that this would have substantially influenced our results since gestational age is highly correlated with birthweight, which was included in all multivariate analyses. There is also a risk of residual confounding due to incomplete adjustment for SES. As described in the methods section, the average income for the postal code of residence was used as the only marker of SES due to incomplete information about parental educational attainment. We have conducted a number of assessments to confirm that this was our most suitable option. Reassuringly, we also found comparable results in the subgroup of families for which we have information on both parental education and average income for the postal code of residence. The use of this a postal code average income as an indicator is also in line with a Swedish study which suggested that neighbourhood purchasing power may be a useful special determinant of breastfeeding rates [32]. However, we acknowledge that there is a risk of non-differential misclassification bias due to the identification of group-level SES, particularly since lower SES appears to be associated with both lower rates of breastfeeding rates [33] and high rates of allergy-related disease [34] in Norway and the Nordic countries. It would be advisable that future studies use both parental educational attainment and family income to further reduce the risk of residual confounding. Studies in other countries may also need to consider other potential confounding factors, such as day-care attendance, which can influence both breastfeeding duration and may influence asthma and wheeze before 6-years of age [35]. Due to the generous parental leave in Norway, day-care attendance before one year of age is uncommon and does not represent a significant confounding factor.

In terms of the risk of selection bias, we know from a non-participant study that the participants in the PACT study are similar to the families who opted not to participate [19]. The breastfeeding rates found in this study are also comparable to a study conducted by the Norwegian Bureau of Statistics in 1998 which found that 80% of infants were still breastfed at six months, yet more than 90% had already started some form of complementary food by six months [33]. Among those who submitted at least one lifestyle questionnaire in the PACT study, around a third were either lost to follow-up before completing the child healthcare questionnaire or missing information on breastfeeding and complementary food introduction. We do not have information about why participants did not complete these follow-up questionnaires. This is probably partially due to volunteer bias [36] and partially due to fluctuating interest in the study among the staff at the large number of community health centres who were responsible to handing out the follow-up questionnaires. The variability in recruitment also means that we do not know exactly how many families were invited to participate and this is another limitation of our study and makes it difficult to estimate the degree of potential selection bias. Nonetheless, despite the small differences seen between included and excluded participants in this study, we do not believe these differences affect our results and we consider the results to be generalizable, at least for other Nordic countries. In terms of generalisability beyond Scandinavia, the biological effects of breastfeeding should theoretically also be generalisable to other high incomes countries. Yet, it is difficult to know how the socio-political context affects the interplay between breastfeeding, allergy-related diseases, and confounding factors such as SES, so the observed relationships may be different in other countries and settings.

Finally, with the high proportion of women breastfeeding beyond six months, it is hard to study the risk for children who were not breastfed or breastfed for a shorter duration. Whilst, the extensive data on duration of breastfeeding and accurate age of first complementary foods are well suited to explore the impact of age at weaning and introduction, we did not have sufficient detail in the questionnaires to consider more nuanced infant feeding variations. For example, we do not have information regarding the extent of breastfeeding when the child was receiving a combination of breast and formula milk, or the quantity of the complementary foods after introduction. For this reason, we recommend cautious interpretation of the results of our sensitivity analysis considering the influence of introducing infant formula before six months.

## Conclusions

We found a protective effect of longer breastfeeding duration on wheeze, but not on asthma, at six years. In addition, we saw a trend for a protective effect of longer breastfeeding on ARC up to school age. It remains unclear if these findings represent a protection against allergy mediated ARC, a prolonged effect of early defence against infection that persists after weaning. Our further analyses showed no conclusive associations between duration of breastfeeding, age at introduction to complementary foods and prevention of asthma, wheeze, ARC and eczema.

## Supplementary Information

**Additional file 1: Supplementary Tables** (Additional File 1.pdf) contains the following supplementary tables: **Table S1.** Relationship between income and other common measures of socio-economic status or behaviours associated with socio-economic status; **Table S2**. Characteristics of included participants, excluded participants and those lost to follow up; **Table S3**. Breastfeeding duration versus age at introduction of complementary foods; **Table S4**. Relation between breastfeeding duration and allergy related diseases when symptomatic children before six months of age are excluded; **Table S5**. Relation between breastfeeding duration with maximum one-year recall and allergy related diseases; **Table S6**. Interaction between duration of breastfeeding and with infant formula and allergy related diseases; **Table S7**. Relation between age at introduction to complementary foods and allergy related diseases when symptomatic children before six months of age are excluded; **Table S8**. Relation between breastfeeding / complementary food introduction overlap and allergy related diseases.

## Data Availability

The datasets generated and/or analysed during the current study are not publicly available due to Norwegian privacy legislation but are available from the corresponding author on reasonable request.

## Abbreviations

aOR: Adjusted odds ratio
ARC: Allergic rhinoconjunctivitis
CI: Confidence interval
ISAAC: International Study of Asthma and Allergies in Childhood
NOK: Norwegian kroner
OR: Odds ratio
PACT: Prevention of Allergy among Children in Trondheim
SD: Standard deviation
SES: Socio-economic status
