# Supplementary material for: Duration of breastfeeding, age at introduction of complementary foods and allergy-related diseases: a prospective cohort study
Source: Int Breastfeed J. 2021 Jan 6;16:5. doi: 10.1186/s13006-020-00352-2 (PMC7789718; doi:10.1186/s13006-020-00352-2)
Supplement: Supplementary file 1 — Additional file 1: Supplementary Tables (Additional File 1.pdf) contains the following supplementary tables: Table S1. Relationship between income and other common measures of socio-economic status or behaviours associated with socio-economic status; Table S2. Characteristics of included participants, excluded participants and those lost to follow up; Table S3. Breastfeeding duration versus age at introduction of complementary foods; Table S4. Relation between breastfeeding duration and allergy related diseases when symptomatic children before six months of age are excluded; Table S5. Relation between breastfeeding duration with maximum one-year recall and allergy related diseases; Table S6. Interaction between duration of breastfeeding and with infant formula and allergy related diseases; Table S7. Relation between age at introduction to complementary foods and allergy related diseases when symptomatic children before six months of age are excluded; Table S8. Relation between breastfeeding / complementary food introduction overlap and allergy related diseases. [file 13006_2020_352_MOESM1_ESM.pdf]

Additional File 1: Supplementary Tables

For manuscript:

Duration of Breastfeeding, Age at Introduction of Solid Foods and Allergy Related Diseases: A Prospective Cohort Study

Louise Ekelund, Inga Gloppen, Torbjørn Øien, Melanie Rae Simpson

Table of contents

Supplementary Table S1. Relationship between income and other common measures of socio-economic status or behaviours associated with socio-economic status 2

Supplementary Table S2. Characteristics of included participants, excluded participants and those lost to follow up... 3

Supplementary Table S3. Breastfeeding duration versus age at introduction of complementary foods 4

Supplementary Table S4. Relation between breastfeeding duration and allergy related diseases when symptomatic children before six months of age are excluded 5

Supplementary Table S5. Relation between breastfeeding duration with maximum one year recall and allergy related diseases 6

Supplementary Table S6. Interaction between duration of breastfeeding and supplementary with infant formula and allergy related diseases... 7

Supplementary Table S7. Relation between age at introduction to complementary foods and allergy related diseases when symptomatic children before six months of age are excluded...... 8

Supplementary Table S8. Relation between breastfeeding / complementary food introduction overlap and allergy related diseases 9

Supplementary Table S1. Relationship between income and other common measures of socio-economic status or behaviours associated with socio-economic status

			Income quartiles based on estimated incomes[a]			
	n[b]	Total	Q1	Q2	Q3	Q4
**Maternal smoking[c], %**	6727	19.5	24.5	22.4	16.1	16.1
**Paternal smoking[c], %**	6395	21.9	27.8	23.8	19.0	18.5
**Maternal or paternal smoking[c], %**	6753	30.2	36.7	33.3	26.1	26.0
**Indoor smoking[c], %**	6756	3.3	4.8	4.2	2.2	2.2
**Mothers education, years, mean**	3018	15.7	15.4	15.2	16.0	15.8
**Fathers education, years, mean**	2991	15.1	14.7	14.6	15.4	15.4
**Maternal age, years, mean**	6771	29.9	29.3	29.6	30.5	30.2
**Homeownership[c], %**	6790	89.2	82.6	91.9	91.8	90.2
**Family allergy, %**	6773	73.6	71.5	74.4	73.0	75.3

a: Income estimated based on average income in postcode of residence with participating families divided into quartiles.
b: The numbers vary because of missing data.
c: Smoking habits and homeownership at any time during the first two years of life.

Supplementary Table S2. Characteristics of included participants, excluded participants and those lost to follow up.

Characteristics	Included participants (n = 6802)		Excluded participants (n = 3703)[a]		Lost to follow up (n = 3094)[b]	
	n[c]		n[c]		n[c]	
**Duration of breastfeeding, months, mean (SD)**	6795	11.0 (5.7)	1345	8.9 (4.9)	3028	10.6 (5.6)
**Breastfeeding ≥ 6 months, n (%)**	6796	5695 (83.8)	1348	1010 (75.0)	3093	2560 (82.8)
**Age at introduction to complementary foods, months, mean (SD)**	3814	4.6 (1.3)	1254	4.5 (1.3)	1468	4.6 (1.2)
**Introduced to solid foods ≥ 6 months of age, n (%)**	3814	996 (26.1)	1254	290 (23.1)	1468	352 (24.0)
**Maternal age at birth, years, mean (SD)**	6771	29.9 (4.5)	2731	29.2 (4.7)	3076	29.5 (4.7)
**Maternal education, n (%)**	3018		886		869	
< 12 years (less than high school)		179 (5.9)		68 (7.7)		54 (6.21)
12-16 years (up to 4 years University)		1535 (50.9)		445 (50.2)		454 (52.2)
> 16 years (more than 4 years University)		1304 (43.2)		373 (42.1)		361 (41.5)
**Mean income[b], NOK, mean (SD)**	6524	254527 (29477)	3298	250843 (32378)	2913	251915 (30694)
**Family history of allergy[c], n (%)**	6773	4987 (73.6)	3676	2467 (67.1)	3081	2263 (73.5)
**Older sibling, n (%)**	4680	2648 (56.6)	3605	1813 (50.3)	1848	1017 (55.0)
**Maternal smoking, n (%)**						
During pregnancy	2336	147 (6.3)	2048	196 (9.6)	835	55 (6.6)
During child's first 2 years	6722	1279 (19.0)	2294	379 (16.5)	3054	640 (20.1)
**Paternal smoking, n (%)**	6395	1402 (21.9)	3377	784 (23.2)	2871	654 (22.8)
**Child exposed to smoke during first 2 years[d], n (%)**	6776	1965 (29.0)	2349	672 (28.6)	3077	940 (30.6)
**Pets[e], n (%)**	6802	1408 (20.7)	3703	475 (12.8)	3094	651 (21.0)
**Birth weight, gram, mean (SD)**	6703	3585 (582)	2341	3570 (586)	3039	3590 (600)
**Birth weight <2500g, n (%)**	6703	244 (3.6)	2341	95 (4.1)	3039	127 (4.2)
**Sex, male, n (%)**	6800	3381 (49.7)	2800	1427 (51.0)	3092	1531 (49.5)
**Lower respiratory tract infection within first year[f], n (%)**	3829	411 (10.7)	1264	139 (11.0)	1475	169 (11.5)
**Antibiotic treatment within first year, n (%)**	4447	919 (20.7)	2286	346 (15.1)	1738	360 (20.7)
**Introduced to formula <6 months of age, n (%)**	6723	1940 (28.9)	1562	640 (41.0)	3051	927 (30.4)

a: Participants which have provided at least one lifestyle questionnaire with characteristic information but have missing information on both breastfeeding duration and age at solid food introduction (n = 2339) or have provided no health questionnaire (n = 1364). See Figure 1.
b: Participants lost to follow up because of missing health questionnaire at six years of age.
c: The numbers vary because of missing data.
d: Yearly income in Norwegian kroner based on postcode.
e: Mother, father or common child with parental reported asthma, ARC or AD.
f: Parental or indoor smoking.
g: Reported cat, dog or other furry pets.
h: Pneumonia or bronchitis.

Supplementary Table S3. Breastfeeding duration versus age at introduction of complementary foods

Duration of any breastfeeding	Age at introduction of complementary foods		Total
	≥ 6 months	< 6 months	
**≥ 6 months**	933	2269	3202
**< 6 months**	60	546	606
**Total**	993	2815	3808

Supplementary Table S4. Relation between breastfeeding duration and allergy related diseases when symptomatic children before six months of age are excluded

	Duration of any breastfeeding (N = 6796)				Odds ratio[a] (95 % CI)	
	**≥ 6 months (n = 5695)**		**< 6 months (n = 1101)**			
Disease and age	**n[b]**	**n (%)**	**n[b]**	**n (%)**	**Crude**	**Adjusted[c]**
Asthma						
**2 years (ever)**	4365	204 (4.7)	801	50 (6.2)	0.74 (0.54 - 1.01)	0.81 (0.57 - 1.15)
**6 years (current)**	2724	94 (3.5)	497	19 (3.8)	0.89 (0.54 - 1.49)	0.97 (0.56 - 1.69)
ARC						
**2 years (ever)**	4033	157 (3.9)	732	55 (7.5)	0.50 (0.36 - 0.69)	0.61 (0.43 - 0.87)
**6 years (ever)**	2541	241 (9.5)	458	58 (12.7)	0.72 (0.53 - 0.98)	0.72 (0.52 - 1.02)
Eczema						
**2 years (ever)**	4255	437 (10.3)	775	72 (9.3)	1.12 (0.86 - 1.45)	1.18 (0.89 - 1.57)
**6 years (current)**	2686	279 (10.4)	489	43 (8.8)	1.20 (0.86 - 1.68)	1.31 (0.90 - 1.90)
**6 years (ever)**	2670	383 (14.3)	487	61 (12.5)	1.17 (0.88 - 1.56)	1.24 (0.91 - 1.71)

a: For duration of any breastfeeding for six months or more versus less than six months.
b: The numbers vary because of missing data.
c: Adjusted for sex, maternal age, mean income, first degree relative with allergy, maternal smoking during the child's first two years and birth weight.

Supplementary Table S5. Relation between breastfeeding duration with maximum one year recall and allergy related diseases

	Duration of any breastfeeding (N = 3859)					
	**≥ 6 months (n = 3196)**		**< 6 months (n = 663)**		**Odds ratio[a] (95 % CI)**	
Disease and age	**n[b]**	**n (%)**	**n[b]**	**n (%)**	**Crude**	**Adjusted[c]**
Asthma						
**2 years (ever)**	2646	152 (5.7)	527	41 (7.8)	0.72 (0.50 - 1.03)	0.84 (0.57 - 1.24)
**6 years (current)**	1981	91 (4.6)	383	15 (3.9)	1.18 (0.68 - 2.06)	1.35 (0.74 - 2.49)
Wheeze						
**2 years (ever)**	2564	619 (24.1)	517	147 (28.4)	0.80 (0.65 - 0.99)	0.94 (0.74 - 1.18)
**6 years (current)**	1959	197 (10.1)	382	50 (13.1)	0.74 (0.53 - 1.03)	0.81 (0.56 - 1.16)
ARC						
**2 years (ever)**	2422	129 (5.3)	484	43 (8.9)	0.58 (0.40 - 0.83)	0.69 (0.46 - 1.02)
**6 years (ever)**	1859	231 (12.4)	356	47 (13.2)	0.93 (0.67 - 1.31)	0.91 (0.63 - 1.31)
Eczema						
**2 years (ever)**	2576	414 (16.1)	510	75 (14.7)	1.11 (0.85 - 1.45)	1.18 (0.89 - 1.58)
**6 years (current)**	1944	266 (13.7)	377	45 (11.9)	1.17 (0.83 - 1.64)	1.11 (0.78 - 1.59)
**6 years (ever)**	1934	380 (19.7)	376	66 (17.6)	1.15 (0.86 - 1.53)	1.15 (0.84 - 1.56)

a: For duration of any breastfeeding for six months or more versus less than six months.
b: The numbers vary because of missing data.
c: Adjusted for sex, maternal age, mean income, first degree relative with allergy, maternal smoking during the child's first two years and birth weight.

Supplementary Table S6. Interaction between duration of breastfeeding and supplementary with infant formula and allergy related diseases

	Duration of breastfeeding						Adjusted odds ratio (95 % CI)	
	≥ 6 mo. breastfeeding without supplementation with formula		≥ 6 mo. breastfeeding supplemented with some formula feeding		< 6 mo. breastfeeding formula before 6 mo.		≥ 6 mo. breastfeeding without supplementation with formula vs less than 6 mo. of any breastfeeding	≥ 6 mo. breastfeeding supplemented with some formula feeding vs less than 6 mo. of any breastfeeding
Disease and age	**n[b]**	**n (%)**	**n[b]**	**n (%)**	**n[b]**	**n (%)**		
Asthma								
**2 years (ever)**	4197	262 (6.2)	877	53 (6.0)	959	82 (8.6)	0.79 (0.59 - 1.05)	0.74 (0.51 - 1.10)
**6 years (current)**	2600	121 (4.7)	506	20 (4.0)	559	32 (5.7)	0.79 (0.51 - 1.23)	0.70 (0.38 - 1.28)
Wheeze								
**2 years (ever)**	4076	1050 (25.8)	850	225 (26.5)	934	284 (30.4)	0.91 (0.77 - 1.09)	0.94 (0.75 – 1.17)
**6 years (current)**	2566	265 (10.3)	504	45 (8.9)	558	76 (13.6)	0.72 (0.53 - 0.97)	0.63 (0.42 - 0.95)
ARC								
**2 years (ever)**	3847	209 (5.4)	790	56 (7.1)	867	86 (9.9)	0.61 (0.46 - 0.82)	0.78 (0.54 – 1.15)
**6 years (ever)**	2413	275 (11.4)	473	72 (15.2)	513	74 (14.4)	0.74 (0.55 - 1.01)	1.04 (0.71 - 1.51)
Eczema								
**2 years (ever)**	4087	685 (16.8)	851	160 (18.8)	931	144 (15.6)	1.13 (0.91 - 1.40)	1.32 (1.02 - 1.73)
**6 years (current)**	2558	359 (14.0)	502	75 (14.9)	550	69 (12.6)	1.12 (0.83 - 1.51)	1.24 (0.85 - 1.79)
**6 years (ever)**	2543	503 (19.8)	501	107 (21.4)	548	97 (17.7)	1.14 (0.88 - 1.48)	1.29 (0.93 - 1.78)

b: The numbers vary because of missing data.
c: Adjusted for sex, maternal age, mean income, first degree relative with allergy, maternal smoking during the child's first two years and birth weight.

Supplementary Table S7. Relation between age at introduction to complementary foods and allergy related diseases when symptomatic children before six months of age are excluded

	Introduction to solid foods (N = 3814)					
	**≥ 6 months (n = 996)**		**< 6 months (n = 2818)**		**Odds ratio[a] (95 % CI)**	
Disease and age	**n[b]**	**n (%)**	**n[b]**	**n (%)**	**Crude**	**Adjusted[c]**
Asthma						
**2 years (ever)**	718	31 (4.3)	1971	91 (4.6)	0.93 (0.61 - 1.41)	0.98 (0.62 - 1.53)
**6 years (current)**	560	23 (4.11)	1482	47 (3.2)	1.31 (0.79 - 2.17)	1.56 (0.91 - 2.69)
ARC						
**2 years (ever)**	668	21 (3.1)	1817	82 (4.5)	0.69 (0.42 - 1.12)	0.78 (0.47 - 1.28)
**6 years (ever)**	526	54 (10.3)	1391	134 (9.6)	1.07 (0.77 - 1.50)	1.24 (0.87 - 1.76)
Eczema						
**2 years (ever)**	695	62 (8.9)	1926	181 (9.4)	0.94 (0.70 - 1.28)	0.99 (0.72 - 1.36)
**6 years (current)**	548	43 (7.9)	1459	147 (10.1)	0.76 (0.53 - 1.08)	0.76 (0.53 - 1.11)
**6 years (ever)**	546	60 (11.0)	1450	204 (14.1)	0.75 (0.56 - 1.02)	0.76 (0.55 - 1.04)

a: For introduction to solid foods at six months or older versus younger than six months.
b: The numbers vary because of missing data.
c: Adjusted for sex, maternal age, mean income, first degree relative with allergy, maternal smoking during the child's first two years and birth weight.

Supplementary Table S8. Relation between breastfeeding / complementary food introduction overlap and allergy related diseases

	Overlap (N = 3485)					
	**≥ 2 months (n = 2837)**		**< 2 months (n = 648)**		**Odds ratio[a] (95 % CI)**	
Disease and age	**n[b]**	**n (%)**	**n[b]**	**n (%)**	**Crude**	**Adjusted[c]**
Asthma						
**2 years (ever)**	2530	145 (5.7)	529	39 (7.4)	0.76 (0.53 - 1.10)	0.85 (0.57 - 1.25)
**6 years (current)**	1676	77 (4.6)	377	14 (3.7)	1.25 (0.70 - 2.23)	1.43 (0.76 - 2.70)
Wheeze						
**2 years (ever)**	2452	603 (24.6)	515	133 (25.8)	0.94 (0.75 - 1.16)	1.09 (0.86 - 1.38)
**6 years (current)**	1656	164 (9.9)	377	48 (12.7)	0.75 (0.53 - 1.06)	0.82 (0.56 - 1.18)
ARC						
**2 years (ever)**	2313	127 (5.5)	489	37 (7.6)	0.71 (0.49 - 1.04)	0.83 (0.55 - 1.24)
**6 years (ever)**	1566	198 (12.6)	348	49 (14.1)	0.88 (0.63 - 1.24)	0.87 (0.61 - 1.26)
Eczema						
**2 years (ever)**	2463	397 (16.1)	512	75 (14.7)	1.12 (0.86 - 1.46)	1.17 (0.88 - 1.56)
**6 years (current)**	1645	224 (13.6)	370	43 (11.6)	1.20 (0.85 - 1.70)	1.14 (0.79 - 1.64)
**6 years (ever)**	1637	323 (19.7)	368	65 (17.7)	1.15 (0.85 - 1.54)	1.14 (0.83 - 1.56)

a: For overlap between breastfeeding and introduction to solid foods for two months or more versus less than two months.
b: The numbers vary because of missing data.
c: Adjusted for sex, maternal age, mean income, first degree relative with allergy, maternal smoking during the child's first two years and birth weight.
